# Primary Uterine Primitive Neuroectodermal Tumour Mistaken for Leiomyosarcoma in an Adolescent Girl: A Very Rare Case With Many Diagnostic and Therapeutic Challenges

**DOI:** 10.7759/cureus.105020

**Published:** 2026-03-11

**Authors:** Indira Prasad, Anamika Meena, Madhu Kumari, Pritanjali Singh, Asiya A Ismail

**Affiliations:** 1 Obstetrics and Gynaecology, All India Institute of Medical Sciences (AIIMS), Patna, IND; 2 Radiology, All India Institute of Medical Sciences (AIIMS), Patna, IND; 3 Pathology and Laboratory Medicine, All India Institute of Medical Sciences (AIIMS), Patna, IND; 4 Radiation Oncology, All India Institute of Medical Sciences (AIIMS), Patna, IND

**Keywords:** adolescent, cd99, ewing sarcoma family, leiomyosarcoma, pnet, primitive neuroectodermal tumour, uterus

## Abstract

Primary uterine primitive neuroectodermal tumours (PNETs) are exceedingly rare and typically reported in postmenopausal adults. We describe a 14-year-old adolescent girl who presented with one month of rapidly progressive abdominal distension, lower abdominal pain and nausea. Imaging (ultrasound, contrast-enhanced (CE) CT, and MRI) revealed a large heterogeneous uterine mass with necrosis, enlarged pelvic nodes and suspected peritoneal and pulmonary deposits; serum lactate dehydrogenase was markedly elevated. Preoperative findings suggested leiomyosarcoma, but the patient developed haemoperitoneum before the planned biopsy and required emergency laparotomy.

The uterine corpus was largely replaced by a friable vascular tumour. A total abdominal hysterectomy with bilateral salpingo-oophorectomy and omentectomy was performed. Histopathology showed a small round cell tumour with rosette formation. Immunohistochemistry (IHC) was strongly positive for CD99 and FLI‑1 and Ki‑67, all confirming PNET of the uterine corpus. Preoperative PET/CT demonstrated multifocal metastatic disease. Postoperatively, she was treated with an Ewing sarcoma‑based chemotherapy protocol (vincristine, doxorubicin, cyclophosphamide/ifosfamide and etoposide (VDC/IE)), but unfortunately, she died 10 months after surgery.

Uterine PNETs may mimic more common uterine sarcomas on imaging and may present at an advanced stage. Morphology can overlap with other small round cell tumours. Therefore, IHC with markers such as CD99 and FLI‑1, and, where feasible, molecular testing for EWSR1 rearrangement, are essential for accurate diagnosis. Management typically requires multimodal therapy involving a combination of surgery, radiotherapy and/or systemic chemotherapy. Ewing sarcoma regimens such as VDC/IE are often used because of the shared biology. While on one hand, among adolescents, fertility preservation poses an additional ethical and therapeutic challenge, on the other hand, rapid, individualised decision‑making is also required to prevent clinical deterioration. This case highlights the diagnostic and therapeutic dilemmas faced, the need to consider PNET in the differential diagnosis of aggressive uterine masses even in adolescents, the important role of ancillary testing like IHC and molecular studies for a definitive diagnosis and the difficulties in balancing life‑saving surgery and fertility preservation when disease is advanced.

## Introduction

Primitive neuroectodermal tumours (PNETs) of the uterus are rare, highly malignant small round cell neoplasms that are considered part of the Ewing sarcoma family of tumours (ESFT). Although PNET most commonly arises in the central nervous system or at osseous and soft-tissue sites, visceral presentations, particularly within the uterine corpus, are uncommon; approximately 90 cases have been reported in the literature [1,2]. Reported patients most often present in adulthood, typically with vaginal bleeding or a pelvic mass, and many present with locally advanced disease.

Diagnosis is challenging because imaging features are non‑specific and histological appearance overlaps with other small round cell tumours such as rhabdomyosarcoma, small cell carcinoma, lymphoma, germ cell tumours and metastatic neuroblastoma. Accurate classification, therefore, relies on integration of morphology, immunohistochemistry (IHC) and, where available, molecular testing. Immunohistochemical markers frequently used to support a diagnosis of ESFT/PNET include membranous CD99 and nuclear FLI‑1, along with neural markers, like molecular confirmation of EWSR1 gene rearrangement, which is diagnostic when present [3,4].

Because uterine PNET is rare, there are no standardised management guidelines. Reported management approaches are multimodal and often extrapolated from ESFT practice, including surgery, systemic chemotherapy and radiotherapy in selected cases. Prognosis is variable but often guarded. Given the paucity of data, detailed case reports and case series remain important to better define diagnostic pathways, optimal treatment strategies and outcomes for this uncommon tumour.

## Case presentation

A 14-year-old schoolgirl presented to our hospital with a four-week history of rapidly progressive abdominal distension, dull lower abdominal pain and nausea. The girl had attained menarche six months back and gave a history of three menstrual cycles prior to presentation to the hospital with this mass. There was no complaint of vaginal bleeding, no significant past medical history and no family history of malignancy. On examination, she appeared pale; abdominal palpation revealed a large, firm, immobile mass consistent with a 24- to 26-week gravid uterus whose lower margin could not be delineated, and on per-rectal examination, nothing significant was found. A single palpable right inguinal lymph node was noted. Ultrasonography and contrast CT performed at an outside facility revealed a large, heterogeneously enhancing pelvic mass arising from the anterior uterine wall, extending to the umbilicus, and causing bilateral hydroureteronephrosis. Multiple enlarged iliac nodes and peritoneal deposits were also noted, but the ovaries were not well visualised. The radiological impression suggested leiomyosarcoma with metastatic disease.

**Figure 1:**
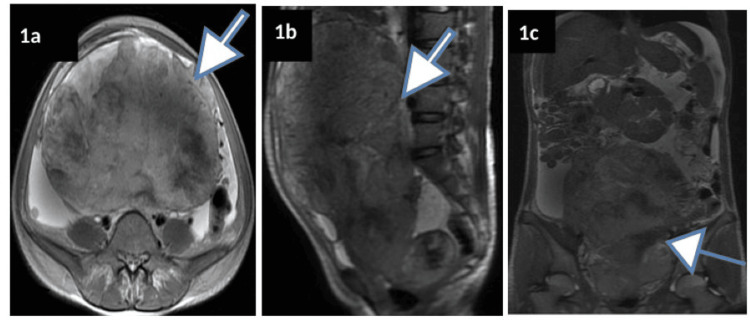
Preoperative MRI revealing a large, relatively well-circumscribed soft tissue mass (arrows) measuring 23 × 18.5 × 12 cm centered in the uterine corpus The mass is well visualised on the axial (a), sagittal (b), and coronal (c) views, highlighting its extent and invasive nature. It exhibits irregular margins and demonstrates infiltration into the myometrium and parametrium while displacing and compressing the adjacent pelvic structures. However, there was no apparent extension to the anterior rectal wall and posterior bladder wall.

**Figure 2:**
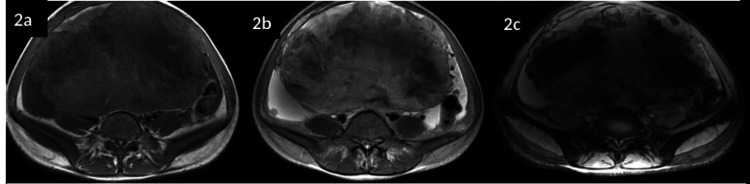
Preoperative MRI signals suggesting possibility of malignancy a: Mass displays an intermediate signal with areas of high signal on T1; b: Inhomogeneous high signal on T2; c: Multiple foci and areas of blooming are seen within the mass on gradient echo (GRE)

**Figure 3:**
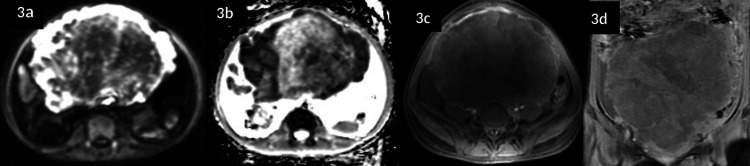
Preoperative diffusion-weighted and post-contrast MRI a and b: Marked peripheral restriction, reflecting high cellularity, with low apparent diffusion coefficient (ADC) values; c and d: Postcontrast sequences that show a heterogeneous mild enhancement with large centro-tumoural necrosis

At our centre, pelvic MRI (T1/T2/contrast + diffusion) demonstrated a 23 × 18.5 × 12 cm ill-defined, heterogeneously enhancing mass centred in the uterine corpus with areas of central necrosis, diffusion restriction, irregular margins infiltrating the myometrium and parametrium, and enlarged pelvic nodes (Figures 1-2). The large mass exhibited irregular margins, infiltration into the myometrium and parametrium, compression of adjacent pelvic structures, and large central necrosis (Figure 3).

**Figure 4:**
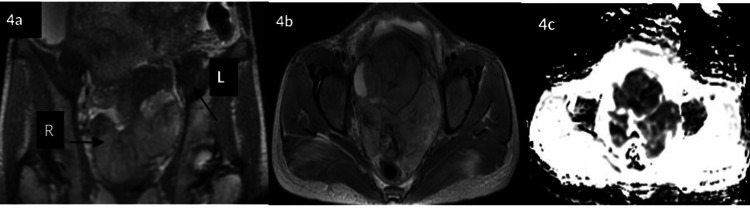
MRI highlighting bulky ovaries The coronal (a) and axial (b) T2 sequence shows bilateral bulky ovaries (black arrows) and blooming foci on axial GRE with mild homogenous enhancement on post-contrast T1 sequence. Small areas of diffusion restriction are visualized in the bilateral ovaries on the diffusion weighted imaging (DWI)-ADC sequence (c), suggesting metastatic involvement. ADC: Apparent diffusion coefficient

**Figure 5:**
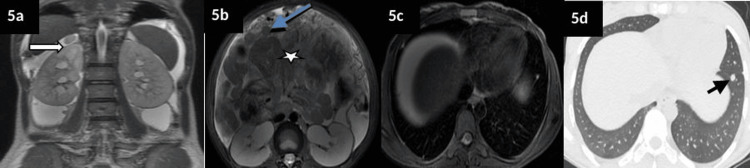
Preoperative MRI and HRCT images with metastatic deposits The coronal (a) and axial (b) T2 sequences showed moderate hydroureteronephrosis seen due to mass effect on either side with moderate ascites with small peritoneal deposits (blue arrow), omental caking (star) and right adrenal nodule (white arrow) along with small lung nodules (black arrow) in visualised lung bases on either side (c and d) of the HRCT lung window. HRCT: High-resolution CT

**Figure 6:**
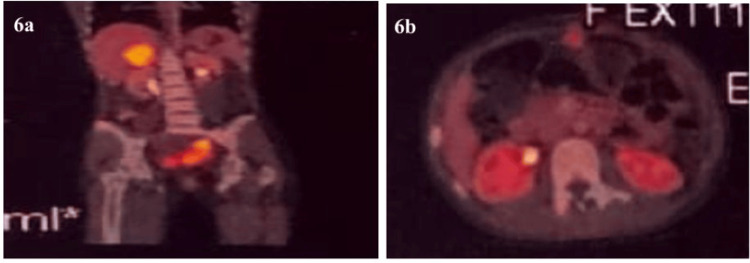
PET/CT images showing metastasis a: Sagittal view of active hypermetabolic areas of metastasis in the liver and spleen; b: Axial view of active hypermetabolic areas of metastasis in the right adrenal gland

Bilateral bulky ovaries could be appreciated separately on the MRI. The right ovary was reported to be cystic and enlarged, while the left ovary appeared normal (Figure 4). Multiple enlarged iliac nodes, peritoneal deposits, deposits on the adrenal gland and mild hydroureteronephrosis were also identified on MRI (Figure 5, panels a-b). While an MRI of the brain was found to be normal, the high-resolution (HR) CT chest and subsequent PET/CT showed multiple bilateral pulmonary nodules and multifocal extra-abdominal deposits (omental, adrenal, bone, and nodal) suspicious for metastases (Figure 5, panels c-d; Figure 6).

The adolescent girl also underwent biochemical evaluation as part of the workup. The laboratory results were notable, with markedly elevated lactate dehydrogenase (LDH) at 2666 U/L (reference range 230-460 U/L) and an elevated serum CA-125 of 154.3 U/mL (reference <35 U/mL). Thyroid-stimulating hormone (TSH) was 4.72 µIU/mL (reference range: 0.35-5.5 µIU/mL), which was within normal limits. Other tumour markers were also within normal limits: serum alpha-fetoprotein (AFP) <1.3 ng/mL (reference range: 0-8.5 ng/mL), carbohydrate antigen (CA) 19-9 0.49 U/mL (reference range: <37 U/mL), carcinoembryonic antigen (CEA) 0.09 ng/mL (reference range: 0-2.5 ng/mL) and serum inhibin 1.2 pg/mL (early follicular reference range: 1.8-17.3 pg/mL). Results also showed that her haemoglobin was 7.1 g/dL, consistent with moderate anaemia per the WHO criteria (7.0-9.9 g/dL).

Initial differentials based on imaging and elevated LDH included leiomyosarcoma and other uterine sarcomas, germ cell tumours, high-grade endometrial stromal sarcoma, metastatic small round cell tumours and PNET/ESFT. Given the patient’s age, germ cell tumours and rhabdomyosarcoma were also considered. Definitive histopathology and IHC (of the biopsy tissue from the lump) were planned to clarify the diagnosis.

**Figure 7:**
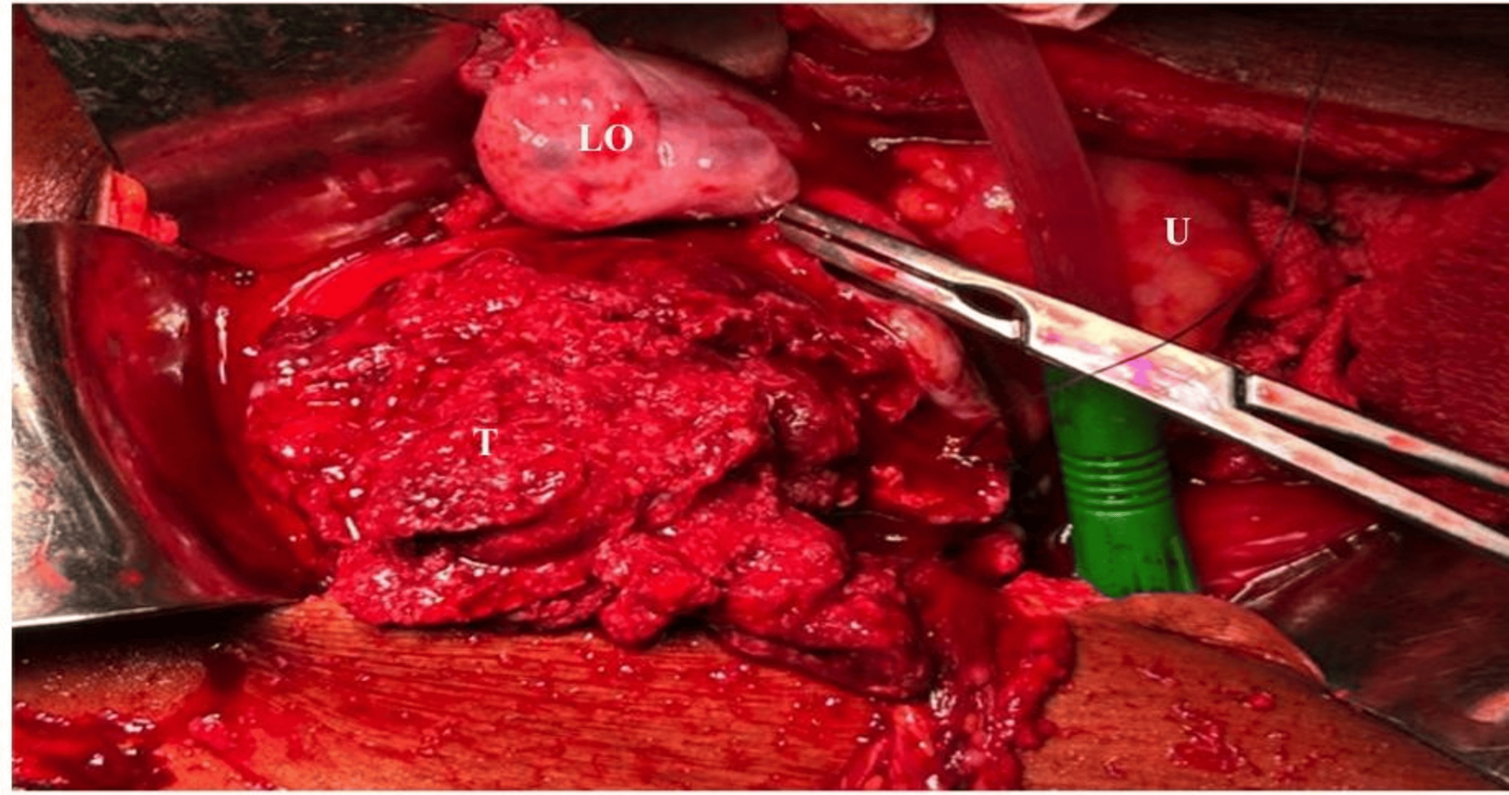
Intraoperative view of the tumour Intraoperative picture of highly distorted, vascular and friable tumour (T) arising from the uterine corpus (U) and the bulky ovary being clamped (LO).

**Figure 8:**
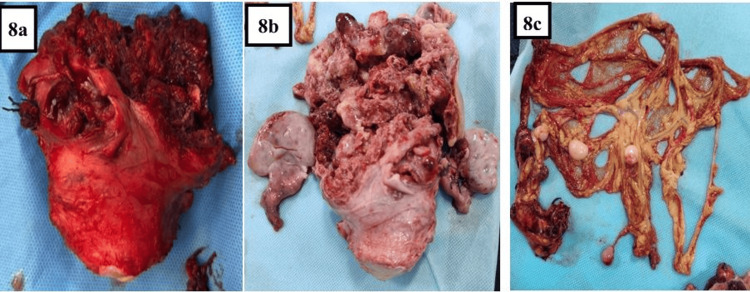
Gross macroscopic view of the tumour a: A partial specimen of the uterus with the tumourous growth arising from the fundus. Most of the friable tumour arising from the uterine body, removed during laparotomy, was sent for frozen section. b: Gross specimen (fixed in formalin) of the removed uterus with a distorted and friable tumour arising from the uterine corpus, bilateral ovaries, and fallopian tubes. c: Omentectomy specimen with omental and peritoneal nodules

An image-guided biopsy was planned to obtain tissue before systemic therapy with the intent to consider fertility-sparing options if feasible. However, on the morning of the procedure, she developed acute abdominal pain with signs of peritonitis. Emergency laparotomy was therefore performed. Operative findings included haemoperitoneum and the entire uterine corpus replaced by a friable, highly vascular mass with areas of tumour rupture (Figure 7). Due to the extent of disease and active bleeding, a decision for complete surgery was taken, and a total abdominal hysterectomy with bilateral salpingo-oophorectomy, omentectomy and repair of a bladder injury was performed (Figure 8). Estimated blood loss was about two litres. One unit of packed red cells was transfused intraoperatively and one unit postoperatively.

**Figure 9:**
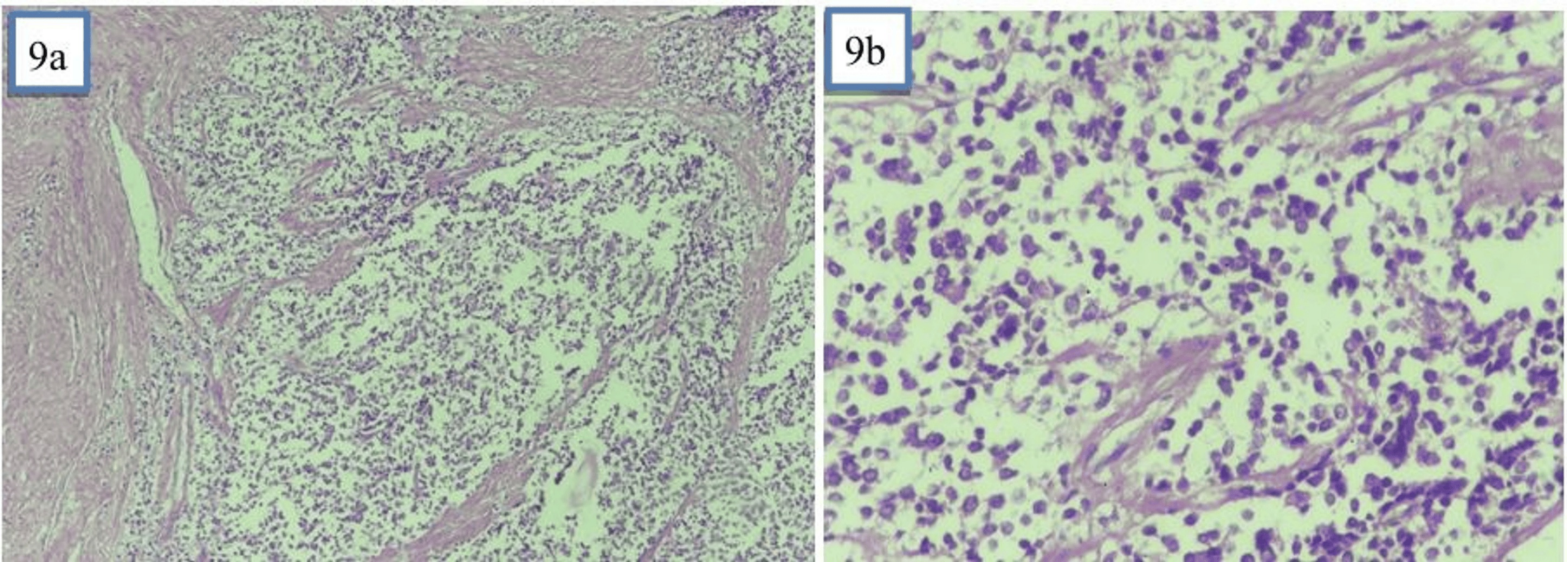
Histopathology of the tumour a: Haematoxylin and eosin (H&E) staining of tumour tissue seen at 10x magnification; b: H&E staining of tumour tissue seen at 40x magnification with rosetting

The intraoperative frozen section described an aggressive malignant neoplasm with suspected carcinosarcoma. Final histopathology reported a poorly circumscribed, infiltrative small round cell tumour arising from the myometrium with sheets of small round cells, rosette formation, frequent necrosis, lymphovascular invasion and metastases to the omentum and 19 lymph nodes with extranodal extension (pT2b, pN1, pMx). These tumour cells, arranged in sheets separated by fibrous septae, were small and round, had stippled nuclear chromatin, inconspicuous to conspicuous nucleoli, and scant amounts of clear to eosinophilic cytoplasm. Rosetting of tumour cells was also identified (Figure 9). Extensive areas of necrosis, lymphovascular invasion and multiple nodal metastases with extranodal extension were noted. The tumour showed aggressive malignant behaviour, with a mitotic rate of 2-3/high-power field (HPF) and atypical mitotic figures. The right ovary, cervix and serosa of the uterine corpus were involved in the tumour.

**Figure 10:**
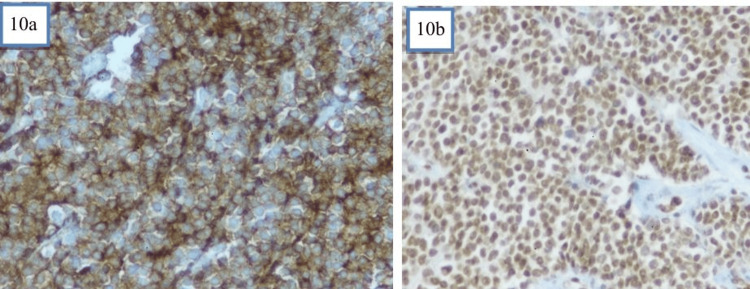
IHC of the tumour a: IHC slide revealing uniform CD99 positivity; b: IHC slide showing FLI-1 positivity;  IHC: Immunohistochemistry

Defying the diagnosis of leiomyosarcoma, a diagnosis of PNET was rendered on histopathology with a recommendation for ancillary IHC for confirmation. Immunohistochemistry revealed strong, diffuse membranous positivity for CD99 and nuclear positivity for FLI-1. The Ki-67 index was 65% to 70% (Figure 10). Markers to exclude other entities (cytokeratin, desmin, myogenin, leukocyte common antigen (LCA), and germ cell markers) were negative or not supportive of alternative diagnoses. These findings supported the diagnosis of PNET (an ESFT) of the uterus.

Postoperatively, the patient recovered and was discharged on postoperative day five. She was started on an Ewing sarcoma-based chemotherapy regimen with palliative intent, given the advanced metastatic status at presentation. The regimen included vincristine, cyclophosphamide and doxorubicin (VDC) and dactinomycin alternating with ifosfamide and etoposide (IE), as used in the INT-0091 protocol. The palliative systemic therapy was initiated with vincristine 1.5 mg/m² IV on day one, doxorubicin 75 mg/m² IV on day two, and cyclophosphamide 1,200 mg/m² IV on day one, alternating with ifosfamide 1,800 mg/m²/day IV on days one to five plus etoposide 100 mg/m²/day IV on days one to five; VDC/IE is given on a 21/28-day cycle. Dosing was calculated per institutional paediatric oncology protocols and adjusted for body surface area. She tolerated treatment reasonably well until the fifth chemotherapy cycle, after which she developed intestinal obstructive features and progressive systemic complications. Despite palliative chemotherapy, she succumbed before the sixth cycle. She survived approximately 10 months from initial presentation: she had noticed abdominal distension four weeks before presenting to the hospital, underwent emergency surgery four days after presentation, and systemic chemotherapy was initiated four weeks after surgery. The timeline of her case is summarised in Table 1. 

**Table 1:** Summary of the case with timeline of significant events featuring clinical findings, imaging findings, pathology results, final diagnosis, and adjuvant chemotherapy IHC: Immunohistochemistry, CECT: Contrast-enhanced CT, HRCT: High-resolution CT

Significant events	Timeline
Clinical red flags (symptoms): Rapid mass growth, vague pain with an increase in severity of pain sometimes, systemic symptoms of weakness, etc. On examination, a firm to hard lump corresponding to a 24- to 26-week gravid uterus was noted.	Four weeks before presentation to the local clinic that advised an abdominal ultrasound
Initial investigations: Pelvic ultrasound transabdominal sonography (TAS)	Took seven days and was referred to a higher centre
Further imaging: CECT abdomen and pelvis	Took another five to seven days. The patient was informed of a poor prognosis and referred again.
Presented to our institute: Pelvic MRI for further characterisation; Laboratory studies: CBC, inflammatory markers, tumour markers	Took 24 hours
The MRI pelvis and HRCT chest suggested metastatic deposits. A PET/CT confirmed metastatic deposits in the adrenal, lungs and lymph nodes.	Took 24 hours
Management: A prompt multidisciplinary discussion with surgical oncology, medical oncology, pathology, and reproductive specialists was conducted with explicit documentation of fertility-preserving versus life-saving priorities	Advised a biopsy from the mass for tissue diagnosis to be followed by chemotherapy. The management plan was decided unanimously.
The patient was planned for an ultrasound-guided biopsy	On the day of the planned biopsy, the patient developed signs of peritonitis. An emergency laparotomy was performed.
Emergency surgery: Exploratory laparotomy with total abdominal hysterectomy with bilateral salpingo-oophorectomy was performed	Frozen section from the operation table suggested a high-grade carcinosarcoma. So, the ovaries were also removed
Complete tissue diagnosis: Pathology workup with a comprehensive IHC panel	Final reporting took almost 15 days, which suggested a primitive neuroendocrine tumour of the uterus.
Adjuvant chemotherapy: INT-0091 protocol	After 28 days of the surgery, the first cycle of chemotherapy was initiated
She succumbed before the sixth cycle	The patient survived for 10 months after her first presentation

## Discussion

This case illustrates several important issues and diagnostic challenges. The timeline of various events significant in this case has been summarised in Table 1. Primitive neuroectodermal tumours are included in the family of Ewing sarcomas. They have a neuroectodermal origin resulting from the translocation t(11;22)(q24;q12), which produces the EWS-FLI1 fusion transcription factor. Most PNETs are located along the central axis, deriving from the neural tube in the brain or spinal cord. The location of PNET in the uterine body is extremely rare, with only approximately 90 reported cases between 1986 and 2021 in the review by Weinstein et al. [1]. These tumours typically affect postmenopausal women who present with vaginal bleeding and advanced-stage disease.

On imaging, uterine PNETs lack pathognomonic features and may be radiologically indistinguishable from more common aggressive uterine sarcomas (e.g., leiomyosarcoma), as both can present as large, heterogeneous necrotic masses with nodal or peritoneal spread and elevated LDH. The MRI defines the extent but not the histological type of the mass. On histopathology, uterine PNET is a small round blue cell tumour that can show rosette formation; such a morphology overlaps with rhabdomyosarcoma, small cell carcinoma, lymphoma and poorly differentiated carcinoma. Hence, a broad IHC panel is essential for confirming the diagnosis. On IHC and molecular testing, diffuse membranous CD99 and nuclear FLI1 positivity support a diagnosis of ESFT/PNET, but are not entirely specific. Molecular confirmation of EWSR1 gene rearrangement by fluorescence in situ hybridisation (FISH) or RT-PCR provides definitive evidence when available and may guide inclusion in Ewing-type protocols.

Multimodal therapy is commonly employed. In most published uterine PNET cases, combinations of radical surgery, systemic chemotherapy and radiotherapy have been described, as in a case report by Blattner et al. [5]. Immunotherapy, including immune checkpoint inhibition, anti‑GD2 strategies, and adoptive/cellular approaches, is under active investigation in sarcoma/Ewing‑family tumours but remains investigational for uterine PNET due to limited clinical evidence. Most literature notes that the standard management continues to rely on surgery, cytotoxic chemotherapy, and radiotherapy for these tumours. However, due to the rarity of uterine PNETs, there is no consensus on management. Sarcoma- or Ewing-based chemotherapy regimens have shown some promise, with higher overall survival observed in patients with stage I disease and those who receive such treatment [6]. Given shared biology, many clinicians apply Ewing sarcoma regimens (VDC/IE) that have proven benefit in classic Ewing sarcoma, as demonstrated in the INT-0091 trial [7]. However, uterine location, older patient age, and frequent advanced stage at presentation complicate management and prognosis.

In adolescents and young adults, fertility preservation is an important concern. For localised disease, fertility-sparing surgery might be considered after careful multidisciplinary discussion. However, when the disease is advanced, life-saving surgery takes priority. In emergent presentations with haemoperitoneum, fertility-sparing options are often not feasible. Most reported uterine PNETs present at an advanced stage and are aggressive with poor long-term outcomes [8,9]. Younger age may be associated with a better short-term response to aggressive chemotherapy in some series, but advanced metastatic disease carries a poor prognosis [1,2,9].

## Conclusions

This case report illustrates that PNETs should be considered in the differential diagnosis of rapidly enlarging uterine masses, even in adolescents. Rapid workup and institution of multimodal management are essential for optimum outcomes in such aggressive cases. Unfortunately, many cases present at an advanced stage, and overall five-year survival remains poor. A definitive diagnosis of PNET requires histopathology supported by IHC (CD99, FLI1) and, where possible, molecular confirmation of EWSR1 rearrangement. Fertility preservation is an important consideration in young patients, but must be balanced against immediate life-threatening complications and disease stage. Further research is needed to understand the genetic and genomic underpinnings of this disease and to identify effective treatment strategies.
